# A Rationalization of the Effect That TMAO, Glycine, and Betaine Exert on the Collapse of Elastin-like Polypeptides

**DOI:** 10.3390/life12020140

**Published:** 2022-01-18

**Authors:** Andrea Pica, Giuseppe Graziano

**Affiliations:** 1 ALPX, 71 Avenue des Martyrs, 38000 Grenoble, France; andrea.pica@alpx-services.com; 2 Dipartimento di Scienze e Tecnologie, Università del Sannio, Via Francesco de Sanctis snc, 82100 Benevento, Italy

**Keywords:** elastin-like polypeptides, solvent-excluded volume effect, coil-to-globule collapse transition, stabilizing co-solutes

## Abstract

Elastin-like polypeptides (ELPs) are soluble in water at low temperature, but, on increasing the temperature, they undergo a reversible and cooperative, coil-to-globule collapse transition. It has been shown that the addition to water of either trimethylamine *N*-oxide (TMAO), glycine, or betaine causes a significant decrease of T(collapse) in the case of a specific ELP. Traditional rationalizations of these phenomena do not work in the present case. We show that an alternative approach, grounded in the magnitude of the solvent-excluded volume effect and its temperature dependence (strictly linked to the translational entropy of solvent and co-solute molecules), is able to rationalize the occurrence of ELP collapse in water on raising the temperature, as well as the T(collapse) lowering caused by the addition to water of either TMAO, glycine, or betaine.

## 1. Introduction

It is well-established that elastin-like polypeptides, ELPs, are soluble in water at low temperature and undergo a temperature-induced, reversible, and cooperative collapse transition, passing from extended, coil conformations to compact, globular ones [1,2,3]. Soon after collapse, aggregation occurs, and T(collapse) practically corresponds to the lower critical solution temperature. In a recent and very interesting study, Cremer and co-workers tried to shed light on the effect that the addition to water of three co-solutes—trimethylamine *N*-oxide (TMAO), glycine, and betaine—has on the collapse temperature of a specific ELP [4]. The latter consists of 120 repeat units of the sequence Val-Pro-Gly-Val-Gly, for a total of 600 residues. Experimental measurements showed that T(collapse) = 28.5 °C in water, and it decreases significantly on raising the concentration of the three co-solutes. In particular, T(collapse) is 10 °C in 1 M glycine, 12.5 °C in 1 M TMAO, and 18.5 °C in 1 M betaine [4]. In other words, the addition to water of either TMAO, glycine, or betaine stabilizes the globule state of ELP. This result can be considered as “expected” because all three co-solutes are stabilizing agents of the native state of globular proteins [5,6], and the globule state of ELP should resemble the native state of globular proteins. To clarify the mechanism of action of such co-solutes, Cremer and co-workers performed both experimental measurements and computer simulations, obtaining the following results: (1) the surface tension of the aqueous solutions increases with respect to that of water on adding glycine and betaine, but it decreases upon TMAO addition (see Figure 3B in [4]); (2) both glycine and betaine molecules prefer to interact with water and are depleted at the ELP surface, whereas TMAO molecules prefer to interact with ELP and are enriched at its surface (see Figure 4 in [4]); (3) FTIR spectra in the OH stretching region indicate that the addition of TMAO and glycine causes a substantial red-shift effect (which should be indicative of stronger intermolecular H-bonds), whereas betaine addition causes essentially no effect (see Figures 5 and 6 in [4]); (4) the tetrahedral order parameter values, determined by means of MD simulations in SPC/E water [7] and using solute-specific force-fields, show that all the considered co-solutes “disrupt rather than strengthen the water tetrahedral H-bonding network” (see Figure 7 in [4]). These results demonstrate unequivocally that there is no correlation between the T(collapse) lowering of ELP, common to all the three co-solutes, and their effect on: (1) the surface tension of the solutions; (2) the accumulation at the ELP surface; and (3) the position of the OH stretching band. A correlation holds solely between the T(collapse) lowering and the disruption of the tetrahedral H-bonding network of water. However, such a correlation does appear strange because, according to the pictorial iceberg scenario of the hydrophobic effect [8], such co-solutes should be “kosmotropes” (i.e., they should increase the tetrahedral water structure), and, in doing so, they should favor the hydrophobic ELP collapse. The results by Cremer and co-workers lead to the conclusion that traditional explanations do not work well in rationalizing the occurrence of ELP collapse on increasing temperature and the effect of the three stabilizing co-solutes on T(collapse). Indeed, Cremer and co-workers suggested a non-classical mechanism: “TMAO stabilizes proteins by acting as a surfactant for the heterogeneous surfaces of folded proteins.” However, surfactants usually destabilize the native state of globular proteins [9].

We have devised an alternative explanation of the temperature-induced, reversible, collapse transition from the ensemble of extended-swollen conformations (i.e., coil macro-state) to the ensemble of compact-globular conformations (i.e., globule macro-state) of smart polymers, such as poly(*N*-isopropylacrylamide) (PNIPAM), in water and aqueous solutions [10,11,12,13,14]. In general, to insert a solute molecule in a liquid, it is necessary to create a cavity because a liquid is a condensed state of the matter and the existing void volume is partitioned in very small pieces [15] that are not suitable to host a solute molecule. The fundamental role is played by the reversible work of cavity creation at a fixed position, ΔG_c_ (i.e., the corresponding Gibbs free energy change) [16,17]. The latter has a purely entropic origin [18] because it is a measure of the decrease in configurational space accessible to liquid molecules caused by cavity creation (i.e., a decrease in translational entropy). Actually, the relevant quantity is not the van der Waals volume of the cavity (i.e., of the molecule to be hosted) but the solvent-excluded volume of the cavity [19]. The latter, by considering a spherical cavity and a liquid of spherical molecules, corresponds to the sphere whose radius is the sum of the cavity van der Waals radius, r_c_, and the radius of the liquid molecules, r_1_. This is the geometric consequence of the fact that, to have a cavity of radius r_c_, the center of liquid molecules can be located at a distance of at most (r_c_ + r_1_) from the cavity center [16]; note that such a reasoning holds also for different shapes of the cavity and liquid molecules. The solvent-excluded volume can be approximated by the solvent-accessible surface area [20] (in water, it is the water-accessible surface area, WASA). The ΔG_c_ magnitude proves to be particularly large in water due to its large number density and the small size of its molecules [21]. This basic fact rationalizes the poor solubility of nonpolar species in water [22]. When the solute molecule is not rigid but can populate different conformations (i.e., a polymer chain), water molecules play an active role. In order to minimize their translational entropy loss, water molecules push the chain to populate compact conformations that produce a solvent-excluded volume effect smaller than that of extended conformations (i.e., the latter have larger WASA) [10,14,19]. Clearly, in order to have a rich and interesting thermodynamic behavior, the polymer has to be soluble in water, such as PNIPAM or ELP, thanks to their good energetic attractions with water molecules (i.e., H-bonds). In fact, on increasing the temperature, the magnitude of the solvent-excluded volume effect in water increases, and a collapse transition occurs at T(collapse) [10]. The fact that ΔG_c_ is an increasing function of temperature in water and aqueous solutions (i.e., it emerged both in classic SPT calculations [19,23] and in computer simulations in atomistic water models [24]) is a consequence of the almost constancy of water density over the 0–100 °C temperature range, which, in turn, comes from the strength of H-bonds with respect to the random thermal energy.

The collapse transition is cooperative, endothermic, and entropy-driven [25], even though polymer chains pass from extended to compact conformations (i.e., a coil-to-globule collapse). Indeed, the entropy increase comes from the gain in translational entropy of water molecules caused by the WASA decrease associated with polymer collapse. Such a theoretical approach has been extended to rationalize the effect of different co-solutes and co-solvents on PNIPAM T(collapse). For instance, the addition of sodium salts to water causes, in general, a density increase that leads to a rise in the magnitude of the solvent-excluded volume effect (the density becomes relevant as a measure of number density) [10]. The expectation would be a general lowering of PNIPAM T(collapse), but the situation is slightly trickier, depending on the strength of anion energetic attractions for the PNIPAM surface with respect to those for water molecules and on the geometric accessibility of the polymer surface (recognizing that the globule state is characterized by chain fluctuations and not solid-like interior packing [10,23]). In general, anions preferring water stabilize the globule state, lowering T(collapse), whereas anions preferring the PNIPAM surface stabilize the coil state, raising T(collapse). In the present study, we would like to apply the same theoretical approach to the collapse transition of ELP to try to provide a coherent rationalization of the effect the addition of either TMAO, glycine, or betaine has on T(collapse).

## 2. Theory Section

The collapse of some ELPs was investigated by means of DSC measurements, showing that the process is reversible, cooperative, and endothermic [26,27]. The average enthalpy change is ΔH(collapse) = 1.6 kJ molres^−1^, and, assuming T(collapse) = 28.5 °C, ΔS(collapse) = 5.3 J K^−1^ molres^−1^ (note that ELP collapse can be described as a phase transition between two macro-states—the coil one, C-state, and the globule one, G-state—so that ΔG(collapse) = 0 at T(collapse); indeed, a pressure–temperature phase diagram has been obtained [26]). These experimental data, despite their relevance, do not provide clues on the molecular origin of the entropy gain driving ELP collapse. The devised statistical thermodynamic approach leads to the following relationships [19]:

ΔH(collapse) = −ΔE_a_ + ΔH_reorg_ (1)

ΔS(collapse) = ΔΔS_x_ − ΔS_conf_ + ΔS_reorg_ (2)

where the two minus signs are a consequence of our original choice to describe the swelling process, to be in line with the description of globular protein unfolding; ΔE_a_ = [E_a_(C-state) − E_a_(G-state) + ΔE(intra)], where E_a_(C-state) and E_a_(G-state) measure the energetic interactions (i.e., both van der Waals attractions and H-bonds) among the C-state or the G-state, respectively, of ELP and the surrounding water and co-solute molecules; ΔE(intra) is the difference in intra-chain energetic interactions between the C-state and the G-state; ΔH_reorg_ is the enthalpy change due to the structural reorganization of water–water H-bonds upon collapse (i.e., many water molecules pass from the hydration shell of ELP to bulk water); and ΔS_reorg_ is the corresponding entropy change. It has been shown by different authors using different theoretical arguments [21,28,29,30,31] that the structural reorganization of water–water H-bonds produces enthalpy and entropy changes that almost exactly compensate each other:

ΔH_reorg_ = T ΔS_reorg_ (3)

This is in line with the experimental finding that there is no relationship between the effect of a co-solute on water structure and its stabilizing or destabilizing action on the native state of globular proteins [32]. It is important to underscore that: (1) ΔH_reorg_ and ΔS_reorg_ are not small quantities, but they do not affect the overall Gibbs free energy change due to enthalpy–entropy compensation; and (2) ΔH_reorg_ and ΔS_reorg_ depend strongly on temperature because a large positive heat capacity change is associated with the structural reorganization of water–water H-bonds [33]. ΔΔS_x_ is the entropy contribution provided by the difference in solvent-excluded volume between the two states, and ΔS_conf_ represents the gain in conformational entropy of the polypeptide chain upon swelling (for more, see below). On these grounds, the transition Gibbs free energy change ΔG_tr_ = −ΔG(collapse) is:

ΔG_tr_ = [ΔG_c_(C) − ΔG_c_(G)] − T·ΔS_conf_ + [E_a_(C) − E_a_(G) + ΔE(intra)]
= ΔΔG_c_ − T·ΔS_conf_ + ΔE_a_ (4)

where [ΔG_c_(C) − ΔG_c_(G)] = −TΔΔS_x_, and ΔG_c_(C) and ΔG_c_(G) represent the reversible work to create, in water or aqueous solutions, a cavity suitable to host the C-state and the G-state, respectively. The ΔΔG_c_ contribution is calculated by means of a simple geometric model: the G-state is a sphere, and the C-state is a prolate spherocylinder having the same V_vdW_ of the sphere and a larger WASA [10,19]. These geometric assumptions are supported by available data. It has been shown that very high hydrostatic pressures (above 2000 atm) favor the G-state, lowering T(collapse) [26,27]. This datum means that there is a difference in volume between the two ELP macro-states, but it is very small and can safely be neglected when performing model calculations at 1 atm. In addition, MD simulations showed that a marked WASA decrease occurs upon collapse of a 90-residue (VPGVG)_18_, and that both swollen and compact conformations are highly hydrated, with almost all the peptide groups involved in H-bonds with water molecules, regardless of ELP conformation [34].

In the present study, an ELP chain of 601 residues in the G-state is modelled as a sphere of radius *a* = 24.5 Å, V_vdW_ = 61,601 Å^3^, and WASA = 8430 Å^2^, whereas the C-state is modelled as a prolate spherocylinder of radius *a* = 12.25 Å, cylindrical length *l* = 114.33 Å, V_vdW_ = 61,601 Å^3^, and WASA = 12,147 Å^2^ (note that, on average, the residue volume in proteins amounts to 102.5 Å^3^ [35]). The G-state and C-state geometric models are representative of the huge number of conformations belonging to the two macro-states and, for this reason, can be considered to be independent of co-solute addition to water. It is important to underscore that the ΔΔG_c_ contribution: (a) is always positive because ΔG_c_ increases with cavity WASA, even though the cavity V_vdW_ is kept fixed [36,37]; and (b) is calculated by means of the analytic formulas provided by classic scaled particle theory (SPT) for spherical and prolate spherocylindrical cavities in a hard sphere fluid mixture (the pressure–volume term is neglected for its smallness at P = 1 atm) [38,39]. A critical role is played by the volume packing density of the hard sphere fluid mixture (i.e., aqueous solutions), ξ_3_ = (π/6) × Σρ_j_ × σ_j_^3^, where ρ_j_ is the number density, in molecules per Å^3^, of species j and σ_j_ is the corresponding hard sphere diameter; ξ_3_ represents the fraction of the total liquid volume occupied by water and co-solute molecules. The physical reliability of classic SPT formulas is well established [21,39,40,41]. Experimental values of the density of water and the considered aqueous solutions of TMAO, glycine, and betaine were used to perform calculations over the 5–35 °C temperature range [42]. Experimental density values need to be used in order to account for the real attractions that exist among solvent and co-solute molecules and to determine the solution density [43,44]. The following effective hard sphere diameters were used and considered to be temperature-independent: (a) σ(H_2_O) = 2.80 Å [45], corresponding to the position of the first maximum in the oxygen–oxygen radial distribution function of water, at room temperature and 1 atm [46]; (b) σ(glycine) = 5.15 Å, which corresponds to the diameter of the sphere having the experimental partial molar volume of glycine in water [47]; (c) σ(TMAO) = 5.40 Å and σ(betaine) = 6.20 Å, which correspond to the diameters of the two spheres possessing the WASA calculated for the two molecules [48]. Even though different criteria were applied to select the effective hard sphere diameters of the three co-solutes, their relative size is correct in view of the molecular structures.

The T·ΔS_conf_ contribution is estimated by considering that each monomer gains a temperature-independent conformational entropy upon swelling:

T·ΔS_conf_ = T·N_res_·ΔS_conf_(res) (5)

where N_res_ = 601 and ΔS_conf_(res) = 4 J K^−1^ molres^−1^, the same value used in all our previous applications of this approach to thermo-responsive polymers (such as PNIPAM) [10,11,12,13,14]. Even though Equation (5) may appear a rough approximation, its validity is supported by the finding that the denaturation entropy change (of which ΔS_conf_ constitutes a large portion) scales linearly with the number of residues in a large set of globular proteins [49,50]. This term is assumed to be independent of co-solute addition to water (i.e., the conformational entropy is an intrinsic property of polymer chains, largely dictated by steric constraints [51]). According to theoretical approaches and computer simulations [52,53,54], an average value for ΔS_conf_(res) of globular proteins would be around 19 J K^−1^molres^−1^. The marked difference between the two numbers is due to the large conformational entropy characterizing the G-state of ELP in comparison to the unique 3D structure of the native state of globular proteins.

Since ΔG_tr_[T(collapse)] = 0, T(collapse) = 28.5 °C in water, and ΔΔG_c_(water) = 1203.4 kJ mol^−1^ at 28.5 °C, it is possible to take advantage of this constraint via Equation (4) and of the T·ΔS_conf_ estimate reported above, fixing:

ΔE_a_(water) = T·ΔS_conf_ − ΔΔG_c_(water) = 725.2 − 1203.4 = −478.2 kJ mol^−1^ (6)

The finding that ΔE_a_(water) is a negative and not-small quantity should not come as a surprise considering that the C-state has a larger WASA than the G-state, and considering the chemical features of the ELP surface (i.e., the possibility to make H-bonds with water molecules). In addition, since ELP collapse is endothermic and Equation (1) is valid, ΔE_a_(water) is expected to be negative. Using the average per residue contribution reported at the beginning of the Theory section, for a 600-residue ELP, ΔH(collapse) ≈ 960 kJ mol^−1^ and, so, ΔH_reorg_ ≈ 480 kJ mol^−1^. The latter large positive number needs an explanation. A marked WASA decrease is associated with ELP collapse [34]; in other words, a marked decrease in hydration shell size occurs and many water molecules return to the bulk (for a 600-residue ELP, the number can be as large as 800–900 water molecules [34]). This is the structural reorganization of water–water H-bonds, and the finding that ΔH_reorg_ ≈ 480 kJ mol^−1^ means that the difference in strength among H-bonds in the hydration shell and those in the bulk water amounts to a fraction of 1 kJ. The ΔE_a_(water) estimate is considered to be temperature independent in view of the limited temperature range considered in this study (5–35 °C) and is enough to analyze ELP collapse in water and aqueous solutions [4]. Note that it is the ΔH_reorg_ term that is to be strongly temperature dependent [33,55]. The ΔE_a_ quantity is expected to be larger in magnitude in aqueous solutions containing TMAO, glycine, and betaine, due to their attractive interactions with the ELP surface. Since the ΔS_conf_ contribution is assumed to be independent of the co-solute presence, and knowing the different T(collapse) values determined by Cremer and co-workers at different co-solute concentrations [4], the above procedure allows us to also obtain reliable ΔE_a_ estimates in aqueous solutions containing TMAO, glycine, and betaine.

**Figure 1:**
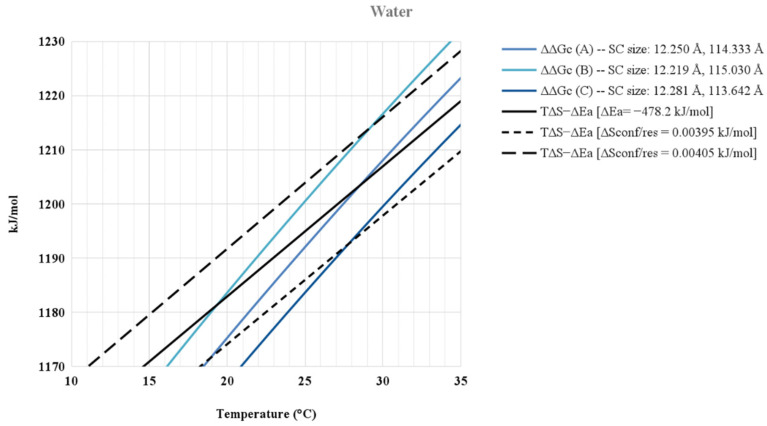
ΔΔG_c_ functions obtained in water by changing the radius and length of the C-state prolate spherocylinder (and keeping fixed the radius of the G-state sphere) and the T·ΔS_conf_ − ΔE_a_ straight lines obtained by considering ΔS_conf_(res) = 4.00 ± 0.05 J K^−1^ molres^−1^. The intersection point represents T(collapse).

An important question is related to the sensitivity of the results to the values assigned to the various parameters of the model. The results are very sensitive to the sizes of the sphere and prolate spherocylinder, and to the value assigned to ΔS_conf_(res) that is multiplied by N_res_ in Equation (5). To highlight such sensitivity, the ΔΔG_c_ functions obtained in water by slightly modifying the radius and length of the C-state prolate spherocylinder (and keeping fixed the radius of the G-state sphere) and the T·ΔS_conf_ − ΔE_a_ straight lines obtained by considering ΔS_conf_(res) = 4.00 ± 0.05 J K^−1^ molres^−1^ (and keeping ΔE_a_ fixed) are shown in Figure 1.

The plot emphasizes the sensitivity and shows that the theoretical approach works well in reproducing the occurrence of ELP collapse around 28 °C, assigning reliable values to the various parameters.

## 3. Results and Discussion

**Figure 2:**
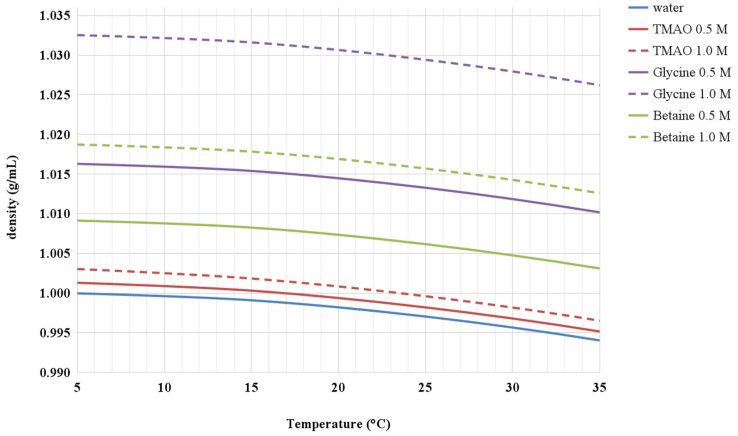
Experimental density of water and 0.5 and 1.0 M TMAO, 0.5 and 1.0 M betaine, 0.5 and 1.0 M glycine aqueous solutions over the 5–35 °C temperature range at 1 atm.

**Figure 3:**
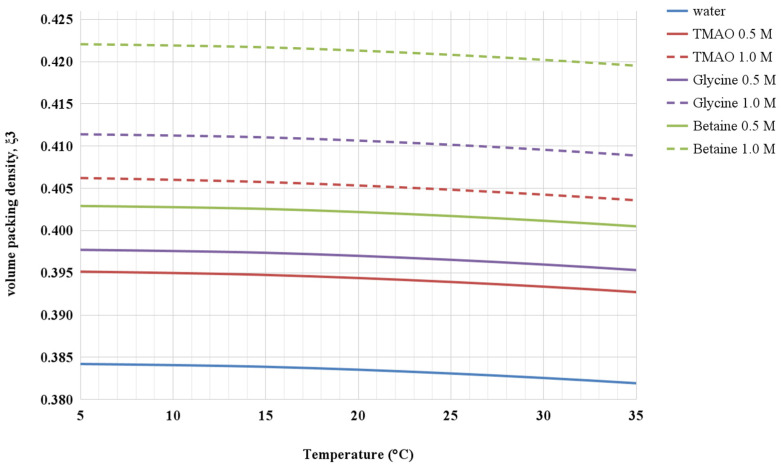
Values of the volume packing density, ξ_3_, for water and for 0.5 and 1.0 M TMAO, 0.5 and 1.0 M betaine, 0.5 and 1.0 M glycine aqueous solutions over the 5–35 °C temperature range at 1 atm.

**Figure 4:**
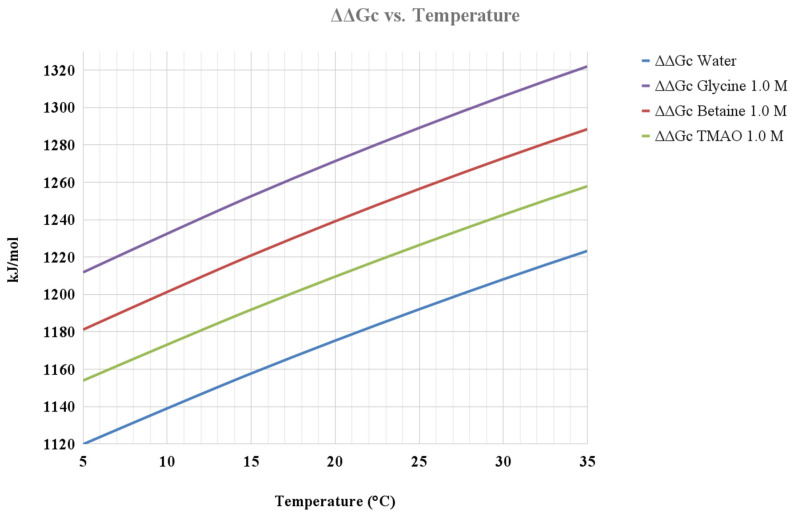
Temperature dependence of the ΔΔGc functions for the ELP, calculated via classic SPT, in water and all the considered aqueous solutions at 1 atm.

Experimental data show that the addition to water of either TMAO, glycine, or betaine causes a density increase that translates into an increase of the volume packing density of the solutions. This is shown in Figure 2 and Figure 3 for the aqueous solutions of the three co-solutes at 0.5 M and 1 M concentrations, in the 5–35 °C temperature range. It is worth noting that, despite the 1 M glycine aqueous solution having the largest density, the 1 M betaine aqueous solution has the largest volume packing density, highlighting the important role of the diameter of co-solute molecules. The corresponding ΔΔG_c_ functions are shown in Figure 4. It is evident that in all the considered aqueous solutions, the ΔΔG_c_ magnitude is larger than that in water (i.e., there is coherence in the effect of the three co-solutes). In all cases, the ΔΔG_c_ function increases with temperature and co-solute concentration, and this occurs to a larger extent in the case of glycine, even though the volume packing density of betaine aqueous solutions is larger. Such a result comes from the basic fact that the diameter of solvent and co-solute molecules has a prevailing role (as already discussed in depth to rationalize the larger ΔG_c_ magnitude in water with respect to that in other liquids [19,22,41]), and glycine molecules are smaller than betaine ones (i.e., the molecular diameter is 5.15 Å versus 6.20 Å, respectively). In general, the ΔΔG_c_ contribution tends to stabilize the G-state, all the more so upon concentration increase of the three co-solutes. It is interesting to note that TMAO, also in the present approach, appears to be special because even though the ΔΔG_c_ magnitude in 0.5 and 1 M TMAO solutions is only slightly larger than that in water, the T(collapse) values are markedly smaller than that in water (see Table 1); this point merits further investigation. In any case, the solvent-excluded volume argument is able to rationalize, in a coherent—though qualitative—manner the experimental finding that the addition to water of either TMAO, glycine, or betaine lowers the T(collapse) value of ELP [4].

To reach a quantitative agreement, it is important to recognize that the stabilizing effect of ΔΔG_c_ is counterbalanced by the destabilizing effect of the ΔE_a_ contribution; this is a large and negative quantity in water, the magnitude of which should rise on adding the three co-solutes because the molecules of the latter can be involved in attractive interactions with the ELP surface [12,13,23]. Robust estimates of the ΔE_a_ contribution are very difficult to obtain using theoretical relationships and/or computational procedures because one would need: (a) reliable ensembles for both the G-state and the C-state of ELP, which is a chain of 600 residues; and (b) good force-fields to describe the interactions of the three co-solutes with both the ELP surface and the water molecules. In contrast, the simple approach outlined to arrive at an estimate of ΔE_a_ at T(collapse) in water (please, see Equation (6)) is feasible and should produce values with internal consistency (any possible error should be more or less of the same entity in all three cases). These ΔE_a_ estimates are listed in the last column of Table 1.

**Figure 5:**
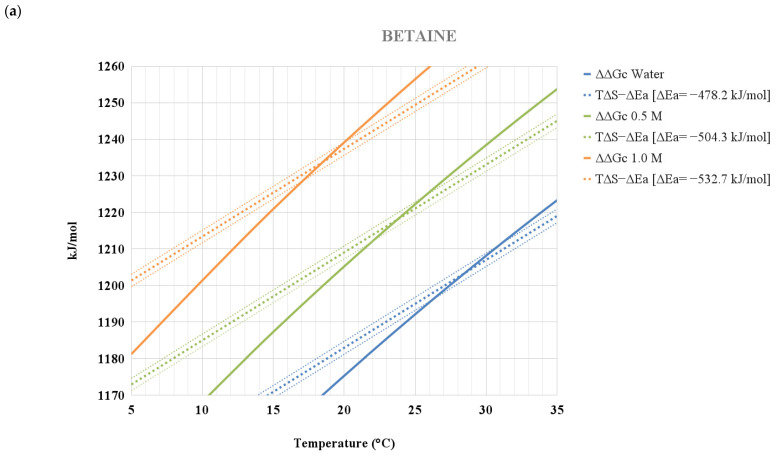

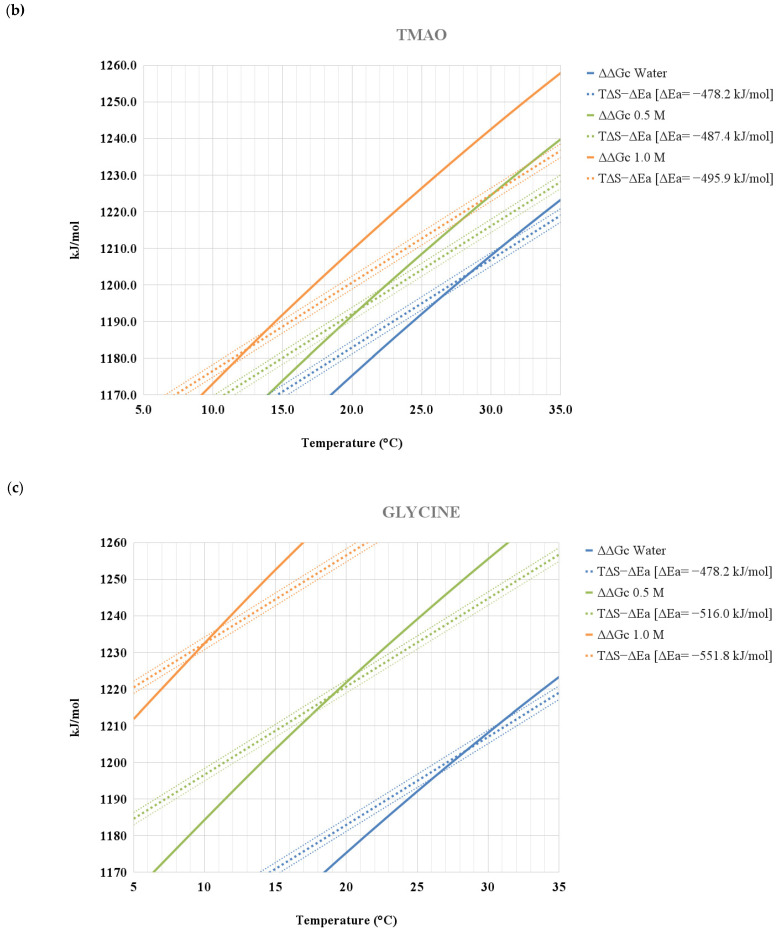
Temperature dependence of the ΔΔGc functions for the ELP: (**a**) in water and in 0.5 and 1.0 M betaine aqueous solutions, together with the corresponding T·ΔSconf − ΔEa straight lines; (**b**) in water and in 0.5 and 1.0 M TMAO aqueous solutions, together with the corresponding T·ΔSconf − ΔEa straight lines; (**c**) in water and in 0.5 and 1.0 M glycine aqueous solutions, together with the corresponding T·ΔSconf − ΔEa straight lines. See the text for further details.

Moreover, they are assumed to be temperature independent in view of the small temperature range over which ELP collapse occurs in the considered aqueous solutions [4] (remember that it is the ΔH_reorg_ term to be strongly temperature dependent [33,55]). This assumption allows the drawing of the T·ΔS_conf_ − ΔE_a_ straight lines that cross the ΔΔG_c_ functions at T(collapse); see Figure 5, panel (a) for betaine aqueous solutions, panel (b) for TMAO aqueous solutions, and panel (c) for glycine aqueous solutions. Actually, the straight lines drawn in Figure 5 also account for a very small uncertainty of 0.01 J K^−1^ molres^−1^, associated with ΔS_conf_(res), to further emphasize the sensitivity of the model results to this parameter. The numbers in the last column of Table 1 indicate that the ΔE_a_ quantity increases in magnitude with the addition of the considered co-solutes to water. This finding makes sense because the molecules of TMAO, glycine, and betaine can all be involved in attractive interactions (i.e., both dispersion interactions and H-bonds) at the ELP surface, and the latter should markedly increase upon swelling of the polypeptide chain. There are quantitative differences among the three co-solutes, but they cannot be taken for granted in view of the simplicity and roughness of the procedure used to arrive at the ΔE_a_ estimates. However, it is important to underscore that preferential interaction (i.e., enrichment) and preferential exclusion (i.e., depletion) are expressions used to describe thermodynamic data referring to differences between conformations belonging to two huge ensembles (i.e., the two macro-states) and cannot be taken literally [56,57,58]. The expectation is that polymer chains possessing both polar and nonpolar moieties, such as PNIPAM and ELP, are attractive for water molecules (indeed, they are soluble in water at low temperature) and for the molecules of TMAO, glycine, and betaine. In fact, MD simulations by Berne and co-workers demonstrated that TMAO molecules, similarly to urea molecules, are enriched at the surface of hydrophobic polymers [59,60]. This reasoning implies that the surface of ELP chains is covered by water and co-solute molecules. Note that the MD results by Cremer and co-workers were obtained not for an ELP chain but for a single Val-Pro-Gly-Val-Gly peptide [4]. To address these matters, it is mandatory to perform MD simulations on polymer chains since the surface area magnitude is a critical factor [61], and additivity might not hold in these cases. Nevertheless, polymer collapse does occur when the translational entropy gain of water and co-solute molecules, associated with the decrease in solvent-excluded volume, overwhelms the other contributions in the Gibbs free energy balance of Equation (4).

In conclusion, the present analysis confirms that the magnitude of the solvent-excluded volume effect and its temperature dependence (strictly linked to the translational entropy of solvent and co-solute molecules) are able to rationalize, in a more than qualitative manner, the occurrence of ELP collapse in water upon raising the temperature. Via the same approach, we rationalize the T(collapse) lowering caused by the addition to water of either TMAO, glycine, or betaine. Approaches grounded in the solvent-excluded volume idea also work well in situations where other approaches fail, and this is something that we would like to highlight.

## Figures and Tables

**Table 1:** Experimental density of water and aqueous solutions of the three co-solutes at 25 °C and 1 atm; volume packing density values at 25 °C; experimental values of T(collapse) for the ELP in the considered solutions from Figure 3A of ref. [4]; classic SPT-ΔΔG_c_ values and T·ΔS_conf_ values at the various T(collapse) values; and estimates of the ΔE_a_ term obtained as in Equation (6). See the text for further details.

	d(25°C) [g ml^−1^]	ξ(25 °C)	T(collapse) [°C]	ΔΔG_c_ [kJ mol^−1^]	T·ΔS_conf_ [kJ mol^−1^]	ΔE_a_ [kJ mol^−1^]
water	0.997	0.383	28.5	1203.4	725.2	−478.2
0.5 M betaine	1.006	0.402	23.5	1218.0	713.7	−504.3
1.0 M betaine	1.016	0.421	18.5	1233.8	701.1	−532.7
0.5 M TMAO	0.998	0.394	20.5	1193.3	705.9	−487.4
1.0 M TMAO	1.000	0.405	12.5	1182.6	686.7	−495.9
0.5 M glycine	1.013	0.397	19.0	1218.3	702.3	−516.0
1.0 M glycine	1.029	0.410	10.0	1232.5	680.7	−551.8

## References

[1] Urry D.W. (1988). Entropic Elastic Processes in Protein Mechanisms. I. Elastic Structure Due to an Inverse Temperature Transition and Elasticity Due to Internal Chain Dynamics. J. Protein Chem..

[2] Nath N., Chilkoti A. (2001). Interfacial Phase Transition of an Environmentally Responsive Elastin Biopolymer Adsorbed on Functionalized Gold Nanoparticles Studied by Colloidal Surface Plasmon Resonance. J. Am. Chem. Soc..

[3] Cho Y., Zhang Y., Christensen T., Sagle L.B., Chilkoti A., Cremer P.S. (2008). Effects of Hofmeister Anions on the Phase Transition Temperature of Elastin-like Polypeptides. J. Phys. Chem. B.

[4] Liao Y.-T., Manson A.C., DeLyser M.R., Noid W.G., Cremer P.S. (2017). Trimethylamine *N* -Oxide Stabilizes Proteins via a Distinct Mechanism Compared with Betaine and Glycine. Proc. Natl. Acad. Sci. USA.

[5] Auton M., Rösgen J., Sinev M., Holthauzen L.M.F., Bolen D.W. (2011). Osmolyte Effects on Protein Stability and Solubility: A Balancing Act between Backbone and Side-Chains. Biophys. Chem..

[6] Record M.T., Guinn E., Pegram L., Capp M. (2013). Introductory Lecture: Interpreting and Predicting Hofmeister Salt Ion and Solute Effects on Biopolymer and Model Processes Using the Solute Partitioning Model. Faraday Discuss..

[7] Berendsen H.J.C., Grigera J.R., Straatsma T.P. (1987). The Missing Term in Effective Pair Potentials. J. Phys. Chem..

[8] Kauzmann W. (1959). Some Factors in the Interpretation of Protein Denaturation. Adv. Protein Chem..

[9] Otzen D. (2011). Protein–Surfactant Interactions: A Tale of Many States. Biochim. Biophys. Acta Proteins Proteom..

[10] Pica A., Graziano G. (2015). On the Effect of Sodium Salts on the Coil-to-Globule Transition of Poly(*N*-Isopropylacrylamide). Phys. Chem. Chem. Phys..

[11] Pica A., Graziano G. (2016). An Alternative Explanation of the Cononsolvency of Poly(*N*-Isopropylacrylamide) in Water-Methanol Solutions. Phys. Chem. Chem. Phys..

[12] Pica A., Graziano G. (2016). On urea’s ability to stabilize the globule state of poly(*N*-Isopropylacrylamide). Phys. Chem. Chem. Phys..

[13] Pica A., Graziano G. (2017). Why Does TMAO Stabilize the Globule State of PNIPAM?. Polymer.

[14] Pica A., Graziano G. (2019). Why Does Urea Have a Different Effect on the Collapse Temperature of PDEAM and PNIPAM?. J. Mol. Liq..

[15] Pohorille A., Pratt L.R. (1990). Cavities in Molecular Liquids and the Theory of Hydrophobic Solubilities. J. Am. Chem. Soc..

[16] Reiss H. (2007). Scaled Particle Methods in the Statistical Thermodynamics of Fluids. Adv. Chem. Phys..

[17] Tomasi J., Persico M. (1994). Molecular Interactions in Solution: An Overview of Methods Based on Continuous Distributions of the Solvent. Chem. Rev..

[18] Lee B. (1985). A Procedure for Calculating Thermodynamic Functions of Cavity Formation from the Pure Solvent Simulation Data. J. Chem. Phys..

[19] Graziano G. (2014). On the Mechanism of Cold Denaturation. Phys. Chem. Chem. Phys..

[20] Lee B., Richards F.M. (1971). The Interpretation of Protein Structures: Estimation of Static Accessibility. J. Mol. Biol..

[21] Graziano G. (2019). Contrasting the Hydration Thermodynamics of Methane and Methanol. Phys. Chem. Chem. Phys..

[22] Graziano G. (2016). Shedding Light on the Hydrophobicity Puzzle. Pure Appl. Chem..

[23] Pica A., Graziano G. (2020). Effect of Sodium Thiocyanate and Sodium Perchlorate on Poly(*N*-Isopropylacrylamide) Collapse. Phys. Chem. Chem. Phys..

[24] Ashbaugh H.S., Pratt L.R. (2007). Contrasting Nonaqueous against Aqueous Solvation on the Basis of Scaled-Particle Theory. J. Phys. Chem. B.

[25] Graziano G. (2000). On the Temperature-Induced Coil to Globule Transition of Poly-N-Isopropylacrylamide in Dilute Aqueous Solutions. Int. J. Biol. Macromol..

[26] Tamura T., Yamaoka T., Kunugi S., Panitch A., Tirrell D.A. (2000). Effects of Temperature and Pressure on the Aggregation Properties of an Engineered Elastin Model Polypeptide in Aqueous Solution. Biomacromolecules.

[27] Yamaoka T., Tamura T., Seto Y., Tada T., Kunugi S., Tirrell D.A. (2003). Mechanism for the Phase Transition of a Genetically Engineered Elastin Model Peptide (VPGIG)_40_ in Aqueous Solution. Biomacromolecules.

[28] Ben-Naim A. (1975). Hydrophobic Interaction and Structural Changes in the Solvent. Biopolymers.

[29] Yu H., Karplus M. (1988). A Thermodynamic Analysis of Solvation. J. Chem. Phys..

[30] Lee B. (1994). Enthalpy-Entropy Compensation in the Thermodynamics of Hydrophobicity. Biophys. Chem..

[31] Dunitz J.D. (1995). Win Some, Lose Some: Enthalpy-Entropy Compensation in Weak Intermolecular Interactions. Chem. Biol..

[32] Batchelor J.D., Olteanu A., Tripathy A., Pielak G.J. (2004). Impact of Protein Denaturants and Stabilizers on Water Structure. J. Am. Chem. Soc..

[33] Graziano G., Lee B. (2005). On the Intactness of Hydrogen Bonds around Nonpolar Solutes Dissolved in Water. J. Phys. Chem. B.

[34] Li B., Alonso D.O.V., Daggett V. (2001). The Molecular Basis for the Inverse Temperature Transition of Elastin11Edited by A. R. Fersht. J. Mol. Biol..

[35] Liquori A.M., Sadun C. (1981). Close Packing of Amino Acid Residues in Globular Proteins: Specific Volume and Site Binding of Water Molecules. Int. J. Biol. Macromol..

[36] Wallqvist A., Berne B.J. (1995). Molecular Dynamics Study of the Dependence of Water Solvation Free Energy on Solute Curvature and Surface Area. J. Phys. Chem..

[37] Graziano G. (2015). The Gibbs Energy Cost of Cavity Creation Depends on Geometry. J. Mol. Liq..

[38] Lebowitz J.L., Helfand E., Praestgaard E. (1965). Scaled Particle Theory of Fluid Mixtures. J. Chem. Phys..

[39] Graziano G. (2011). Contrasting the Denaturing Effect of Guanidinium Chloride with the Stabilizing Effect of Guanidinium Sulfate. Phys. Chem. Chem. Phys..

[40] Graziano G. (2003). On the Cavity Size Distribution in Water and N-Hexane. Biophys. Chem..

[41] Graziano G. (2006). Scaled Particle Theory Study of the Length Scale Dependence of Cavity Thermodynamics in Different Liquids. J. Phys. Chem. B.

[42] Apelblat A. (2016). A New Two-Parameter Equation for Correlation and Prediction of Densities as a Function of Concentration and Temperature in Binary Aqueous Solutions. J. Mol. Liq..

[43] Graziano G. (2012). How Does Sucrose Stabilize the Native State of Globular Proteins?. Int. J. Biol. Macromol..

[44] Cozzolino S., Oliva R., Graziano G., del Vecchio P. (2018). Counteraction of Denaturant-Induced Protein Unfolding Is a General Property of Stabilizing Agents. Phys. Chem. Chem. Phys..

[45] Graziano G. (2008). Salting out of Methane by Sodium Chloride: A Scaled Particle Theory Study. J. Chem. Phys..

[46] Sorenson J.M., Hura G., Glaeser R.M., Head-Gordon T. (2000). What Can X-Ray Scattering Tell Us about the Radial Distribution Functions of Water?. J. Chem. Phys..

[47] Harpaz Y., Gerstein M., Chothia C. (1994). Volume Changes on Protein Folding. Structure.

[48] Street T.O., Bolen D.W., Rose G.D. (2006). A Molecular Mechanism for Osmolyte-Induced Protein Stability. Proc. Natl. Acad. Sci. USA.

[49] Robertson A.D., Murphy K.P. (1997). Protein Structure and the Energetics of Protein Stability. Chem. Rev..

[50] Sawle L., Ghosh K. (2011). How Do Thermophilic Proteins and Proteomes Withstand High Temperature?. Biophys. J..

[51] Zhou A.Q., O’Hern C.S., Regan L. (2011). Revisiting the Ramachandran Plot from a New Angle. Protein Sci..

[52] Baxa M.C., Haddadian E.J., Jumper J.M., Freed K.F., Sosnick T.R. (2014). Loss of Conformational Entropy in Protein Folding Calculated Using Realistic Ensembles and Its Implications for NMR-Based Calculations. Proc. Natl. Acad. Sci. USA.

[53] Sharp K.A., O’Brien E., Kasinath V., Wand A.J. (2015). On the Relationship between NMR-Derived Amide Order Parameters and Protein Backbone Entropy Changes. Proteins Struct. Funct. Bioinform..

[54] Fogolari F., Corazza A., Fortuna S., Soler M.A., VanSchouwen B., Brancolini G., Corni S., Melacini G., Esposito G. (2015). Distance-Based Configurational Entropy of Proteins from Molecular Dynamics Simulations. PLoS ONE.

[55] Pica A., Graziano G. (2016). Shedding Light on the Extra Thermal Stability of Thermophilic Proteins. Biopolymers.

[56] Schellman J.A. (2003). Protein Stability in Mixed Solvents: A Balance of Contact Interaction and Excluded Volume. Biophys. J..

[57] Mukherjee M., Mondal J. (2020). Bottom-Up View of the Mechanism of Action of Protein-Stabilizing Osmolytes. J. Phys. Chem. B.

[58] Martínez L., Shimizu S. (2017). Molecular Interpretation of Preferential Interactions in Protein Solvation: A Solvent-Shell Perspective by Means of Minimum-Distance Distribution Functions. J. Chem. Theory Comput..

[59] Mondal J., Stirnemann G., Berne B.J. (2013). When Does Trimethylamine *N* -Oxide Fold a Polymer Chain and Urea Unfold It?. J. Phys. Chem. B.

[60] Mondal J., Halverson D., Li I.T.S., Stirnemann G., Walker G.C., Berne B.J. (2015). How Osmolytes Influence Hydrophobic Polymer Conformations: A Unified View from Experiment and Theory. Proc. Natl. Acad. Sci. USA.

[61] Rogers B.A., Okur H.I., Yan C., Yang T., Heyda J., Cremer P.S. (2022). Weakly Hydrated Anions Bind to Polymers but Not Monomers in Aqueous Solutions. Nat. Chem..

